# Salt and Temperature Effects on Xanthan Gum Polysaccharide in Aqueous Solutions

**DOI:** 10.3390/ijms25010490

**Published:** 2023-12-29

**Authors:** Emmanuel M. Nsengiyumva, Mark P. Heitz, Paschalis Alexandridis

**Affiliations:** 1 Department of Chemical and Biological Engineering, University at Buffalo, The State University of New York (SUNY), Buffalo, NY 14260, USA; ensengiy@buffalo.edu; 2 Department of Chemistry and Biochemistry, The State University of New York (SUNY) Brockport, Brockport, NY 14420, USA

**Keywords:** biopolymer, polysaccharide, polyelectrolyte, xanthan gum, intrinsic viscosity, hydrodynamic radius

## Abstract

Xanthan gum (XG) is a carbohydrate polymer with anionic properties that is widely used as a rheology modifier in various applications, including foods and petroleum extraction. The aim was to investigate the effect of Na^+^, K^+^, and Ca^2+^ on the physicochemical properties of XG in an aqueous solution as a function of temperature. Huggins, Kraemer, and Rao models were applied to determine intrinsic viscosity, [*η*], by fitting the relative viscosity (*η*_rel_) or specific viscosity (*η*_sp_) of XG/water and XG/salt/water solutions. With increasing temperature in water, Rao 1 gave [*η*] the closest to the Huggins and Kraemer values. In water, [*η*] was more sensitive to temperature increase (~30% increase in [*η*], 20–50 °C) compared to salt solutions (~15–25% increase). At a constant temperature, salt counterions screened the XG side-chain-charged groups and decreased [*η*] by up to 60% over 0.05–100 mM salt. Overall, Ca^2+^ was much more effective than the monovalent cations in screening charge. As the salt valency and concentration increased, the XG coil radius decreased, making evident the effect of shielding the intramolecular and intermolecular XG anionic charge. The reduction in repulsive forces caused XG structural contraction. Further, higher temperatures led to chain expansion that facilitated increased intermolecular interactions, which worked against the salt effect.

## 1. Introduction

Polysaccharide biopolymers are the most significant component of biomass, comprising 90% of the carbohydrate mass in nature [1]. These carbohydrate polymers contain many monosaccharide units linked by α-glycosidic or β-glycosidic bonds, depending on the type of polysaccharide. Most polysaccharides are water-soluble polymers used extensively in many industries as food additives, flocculants, for petroleum extraction, etc. Among polysaccharides, xanthan gum finds many applications as an emulsifier, thickener, and stabilizer agent because of its favorable physicochemical properties [2,3]. XG in aqueous solution is extensively used in the formulation of food, pharmaceutics, cosmetics, and in oil/gas extraction, where salt and temperature vary in the system. While there are literature reports that address XG solution characteristics such as solution viscosity and density, more specific details are needed that compare solution modifiers that include cation identity, salt concentrations, and temperatures in bulk aqueous solutions. Thus, for use in practical applications, it is essential to fully investigate the potentially wide-ranging influence these parameters have on the physicochemical behavior of XG aqueous solutions.

**Scheme 1:**
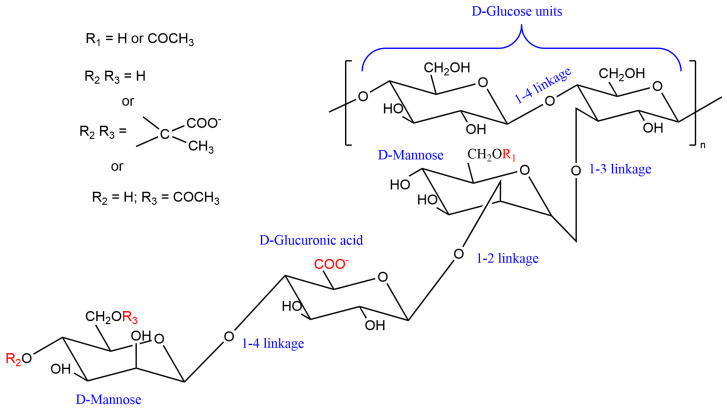
Chemical structure of xanthan gum.

XG contains multiple anionic groups on each side chain, and as a result, it is soluble in water and brines. The XG macromolecule is a strongly water-soluble polymer with a high molecular weight ranging from 1 to 50 × 10^6^ g mol^−1^. XG (Scheme 1) is an extracellular polysaccharide produced from a fermentation process of the Gram-negative bacterium *Xanthomonas campestris* [3].

The primary structure of XG [4,5] and the type of substitution at the outer mannose unit affects the stability of XG conformation. The chemical structure of XG comprises a linear (1 → 4) linked β-D-glucose backbone with a pendant trisaccharide side chain attached to every second glucose unit at the C3 position [5]. The xanthan macromolecule side chains have two mannose residues, one of which may contain either the carboxylic or acetyl group and one the glucuronic acid residue containing the pyruvate group. The content of the acetyl and pyruvate functional groups of xanthan gum chains are affected by bacterial strain and fermentation processes [6]. These functional groups make xanthan gum an anionic polysaccharide [7]. In a salt-containing solution, the side chains wrap around the backbone of the chains, preventing the β-(1-4) linkage from breaking.

When a xanthan gum macromolecule assumes a random coil structure, the dissociation of the side chains from the backbone allows XG chains to interact with each other or other polymer chains, which leads to the formation of a network structure [8]. The network structure forms because of the contribution of intermolecular and electrostatic interactions [6]. However, XG solution structure depends on various factors such as pH, temperature, salt concentration, and others. Brunchi et al. [9] investigated XG viscosity (molecular weight 1.165 × 10^6^ g/mol, 0–0.04 g/dL, at 25 °C) in 0.01–300 mM NaCl or KCl in water. They found small differences (~10%) between the [*η*] values in water and low salt concentrations because the repulsion forces were still effective. The increased Na^+^ and K^+^ cation concentrations screen the charged groups on XG chains in aqueous solutions, and as the screening of electrical charges continues, the XG chains shrink. The screening effect reduces the electrostatic repulsion between charged groups and leads to a more compact conformation and reduction in the hydrodynamic volume of the XG macromolecule. The [*η*] was determined using semi-empirical equations proposed by Rao and Wolf and yielded more accurate results than the Huggins model. The [*η*] decreased by ~74% and ~75% in the presence of 300 mM NaCl and KCl solution compared to water because of the enhancement of the non-Coulombic interactions [9].

Higiro et al. investigated XG (0–0.04 g/dL, no molecular weight reported) and XG/locust bean gum in an aqueous solution at 20 °C [10]. They compared the [*η*] values from the Huggins, Kraemer, Tanglertpaibul, and Rao models. The increasing [*η*] above 306 dL/g was attributed to the dialysis process, which facilitates the removal of inherent impurities with molecular weights smaller than 10 kDa, promotes molecular chain expansion and therefore enhances chain interactions. Their study also used a power-law equation to predict molecular conformation and found a slope of 0.786 associated with a rod-like conformation [10]. Bak et al. studied with viscometry 0.1% *w*/*v* XG (no molecular weight reported) in 0–0.9% NaCl at 25 °C and found a reduction at the high salt concentration (0.9% NaCl) by 47% in the solution viscosity, which they attributed to NaCl-screened charged groups on the backbone of XG chains [11]. They determined the [*η*] values using the Huggins, Kraemer, Tanglertpaibul, and Rao models. They observed the sharpest decrease in the [*η*]~30% NaCl solution compared to salt-free solution because of the charge screening of electrostatic repulsions of the trisaccharide side chain [11].

Banerjee et al. investigated XG (0.01–0.2% at 25–65 °C) in 1000 mM NaCl, HCOOK, CH_3_COONa, (NH_4_)_2_SO_4_, CaCl_2_, and MgSO_4_ [12]. They found that an increment of 30 K in the temperature caused the [*η*] of the XG solution to decrease by ~53%, followed by overall smooth trends with temperature. Monovalent ions showed a comparable effect on the viscosity of the XG solution at 65 °C. For example, the [*η*] decreased by ~6% when comparing 1000 mM NaCl and CH_3_COONa solutions. Their study also reported that divalent ions decreased viscosity more, but their effects were nearly comparable (e.g., smaller by ~5.7% when comparing 1000 mM CaCl_2_ and MgSO_4_ solutions) at 65 °C. The Banerjee work used Huggins, Kraemer, and Arrhenius models to assess the viscosity of the XG solutions [12]. However, only a single salt concentration was considered, whereas, to develop a more robust database of XG solution behavior characteristics, more data is needed to fully understand the concentration landscape. In water, Brunchi et al. reported that the kinematic viscosity of XG (1.16 × 10^6^ g/mol, 0.002–0.02 g/dL at 20–45 °C) decreased with increasing temperature, 20–45 °C [13]. The reduced viscosity slightly decreased with temperature and increased above 33–38 °C, leading to a high increase in the solution viscosity. This suggested the conformation transition of XG chains from an ordered state to a disordered state [13]. Khouryieh et al. showed that the [*η*] of XG (Mw = 2.65 × 10^6^ g/mol, 0.1% *w*/*v*) in water increased with increasing temperature from 154 dL/g at 25 °C to 174 dL/g at 80 °C [14]. Their study used the Huggins, Kraemer, Tanglertpaibul, and Rao models to obtain the [*η*] values.

Xanthan gum has been reported to present a helical, ordered structure at room temperature in aqueous solution or in solutions of low salt concentration, typically less than ~1 mM [3]. An ordered conformation is synonymous with a structure that contains rigid chains and one that produces a solution viscosity less than about 10 mPa·s [5]. In contrast, a disordered XG structure is one in which there is flexibility in the chains and the resulting solution is characterized by a high viscosity, 100–1000 mPa·s. A solution that has increased viscosity is one that is formed by either decreasing salt content or increasing temperature, or both, depending on the extent of pyruvate substitution [6]. Although there are literature reports discussing experimental conditions under which the transition may be observed, it is our view that specific experimental conditions remain elusive as to the particulars of which parameter(s) drive(s) the helix-to-coil transition and under what ranges does the transition occur. Thus, the underlying hypothesis that has governed this work is the question of XG helix-to-coil structural transformation. We evaluated parameters of temperature and ionic strength to study which may be more impactful to XG solution behavior. The practical application of XG occurs in various industries, including food, pharmaceutical, and oil and gas extraction, notably where salinity and temperature can fluctuate [15,16,17]. Thus, studying how these factors affect XG’s performance can help optimize product formulation and process design. Viscosity measurements can provide clues that help to define the structural change; so in this work, we address the following open questions: (1) to what extent do the variables of temperature, XG concentration, and salt concentration collaborate to modify XG solution properties; (2) what is the role of cation identity in influencing the alteration of solution viscosity; and (3) to what extent does temperature impact the water- and salt-based solution viscosity? We have undertaken a systematic approach to investigating these questions to produce a comprehensive study for XG in water, monovalent salt, and divalent salt solutions with varying concentrations and temperatures. Brunchi et al. investigated the XG [*η*] solution in 0.01–100 mM NaCl and 0.01–50 mM KCl solutions at 25–75 °C [9,18]. Banerjee et al. studied the [*η*] of XG in 1000 mM NaCl, CH_3_COONa, (NH_4_)_2_SO_4_, CaCl_2_, and MgSO_4_ at 25–65 °C [12]. Bak et al. examined the intrinsic viscosity of XG in 0–0.9% NaCl solutions at 25 °C [11]. To the best of our knowledge, these few papers describe some of the solution properties, but besides these four articles, there seems to be no other work specifically focused on addressing the effects of salts and temperatures on XG solutions, hence the novelty of the new results presented here.

## 2. Results

### 2.1. Xanthan Gum in Water

**Figure 1:**
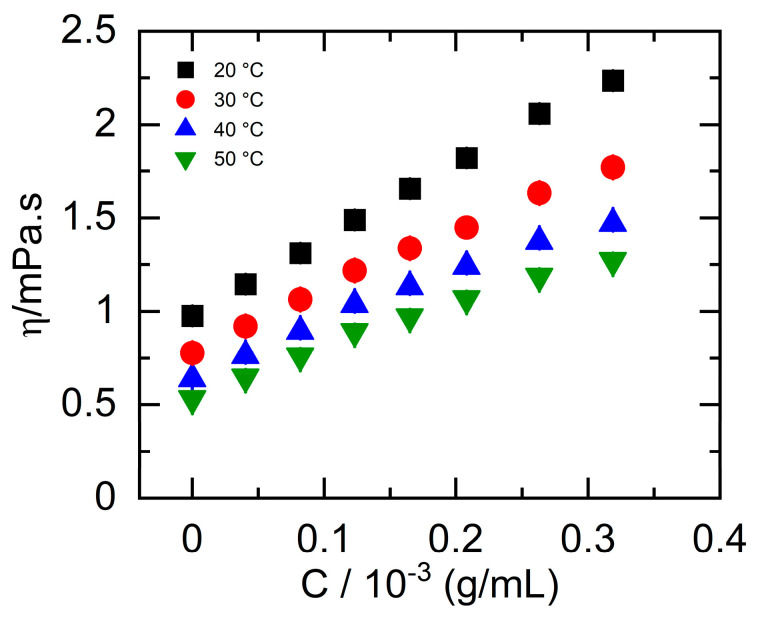
Measured dynamic viscosity as a function of XG concentration in water. The error bars are from relative standard deviations and within the symbols’ size.

**Figure 2:**
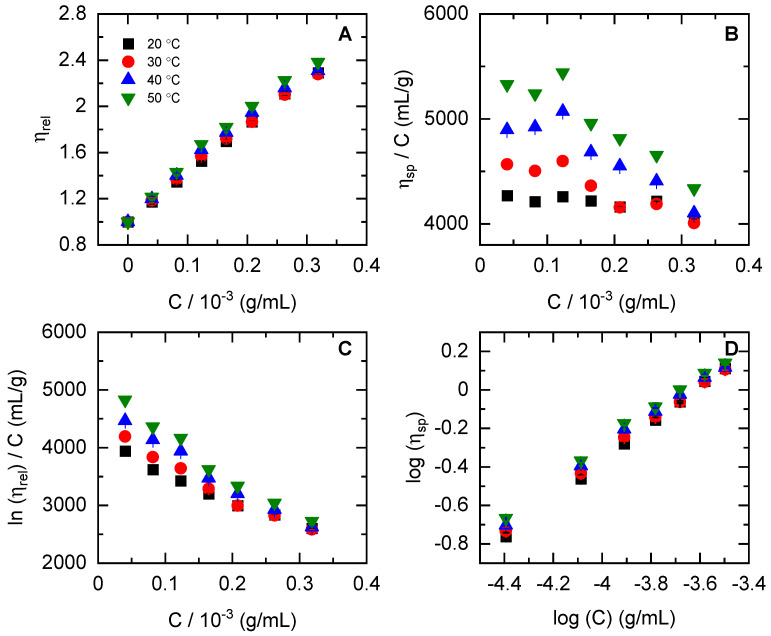
XG concentration dependence of zero-shear viscosity in water for: (**A**) relative viscosity (*η*_rel_, Equation (4)); (**B**) reduced viscosity (*η*_sp_/C, Equation (3)); (**C**) inherent viscosity (ln(*η*_rel_)/*C*, Equation (2)); and (**D**) specific viscosity (log(*η*_sp_), Equation (7)). In all panels, black symbols (■) are 20 °C, red symbols (●) 30 °C, blue symbols (▲) = 40 °C, and green symbols (▼) = 50 °C. The error bars are from relative standard deviations and within the symbols’ size.

To establish a baseline in this work, we first measured the XG aqueous solution zero-shear viscosity by varying the XG concentration, from which we computed relative viscosity (*η*_rel_), reduced viscosity (*η*_sp_/C), inherent viscosity (ln(*η*_rel_)/C), and specific viscosity (log(*η*_sp_)). The zero-shear viscosity data and subsequent calculated viscosities are shown in Figure 1 and Figure 2, respectively. In a salt-free water solution, one expects that the relative viscosity should increase with an increased XG concentration because electrostatic repulsions between charged side chains drive an increase in the polymer hydrodynamic volume [8,9,13,18]. We observed that relative viscosity indeed followed this pattern and that XG showed polyelectrolyte characteristics because of these repulsive Coulombic interactions between charged groups on the side chains. Thus, the reduced viscosity decreased with added XG, particularly at *T* > 30 °C and for XG > 0.0001 g/mL. The reduced viscosity diminution at low [XG] elucidates the expanded conformation of the chains [18]. Two other reasons why the reduced viscosity increased with dilution are as follows: (a) hydrodynamic volume increases when XG is diluted with water because the counterion electrostatic shielding effect is reduced; and (b) fixed ionic charges on the XG contribute to intermolecular osmotic pressure and solvation effects. The value of [*η*] from our data was computed to be 4100 mL/g in water at 20 °C, which we assessed against various literature data points. We found that our value was 28 and 12% smaller compared to [*η*] values for XG reported by Khouryieh et al. [14] and Bak et al. [11], respectively. Compared to other polysaccharides in water, our [*η*] was in several cases much larger, such as with two data points from Potier et al. for alginate and carboxymethyl cellulose, 600% and 300%, respectively [19], 400% for basil seed gum [20], 200% with guar gum [14], and 400% for lepidium perfoliatum seed gum [21]. In comparison to the [*η*] for K-carrageenan [22], the [*η*] for XG is quite similar, with less than 10% deviation. Our [*η*] result is also similar, ~10%, to that obtained for quaternary ammonium salt of curdlan in water [23].

Modeling specific viscosity data using the power-law slope (Equation (7)) can lead to a simple evaluation of the XG conformation in water. It has been reported that slope values < 1 are associated with a rod-like XG conformation, whereas values > 1 are expected for random coil conformations [10]. From our data, a *b*-value of 0.98 was computed for XG in water, and while we might be tempted to assign a rod-like conformation to XG, from viscosity alone one cannot make explicit structural conclusions. Slope values for remaining temperatures are summarized in Table 1.

Comparison to other gums shows varying conformational behavior. For example, in a dilute solution of hsian-tsao leaf gum, Lai and colleagues [24] noted slope values ~0.8, and for a semi-dilute solution, ~1.7, which clearly shows the gum concentration effect and suggests a conformational transition on concentration increase. Higiro and colleagues reported a slope of 0.79 for XG at 20 °C due to interchain electrostatic repulsion [10]. Our XG slope is about 20% higher than both the Lai and Higiro results, implying that, in our solutions, the XG is likely comprised of semi-rigid chains intermediate between rigid rod-like and random coil conformation [25]. For XG and guar gum polysaccharides in dilute solutions, slope values > 1 have been attributed to random coil conformation [12] or even structured entanglements, assuming that the polysaccharide concentration is greater than the overlap concentration [26], which, again, indicate the presence of conformational transitions that are concentration-dependent.

As mentioned in the analysis section, several parameters can be computed from the measured zero-shear viscosity data. When the polymer concentration is increased, the transition from dilute to semi-dilute regimes is characterized by an overlap concentration, C*. This critical concentration is the polymer concentration at which the individual chains begin physically interacting in the solution [8]. We determined C* using Equation (10) and found a value of 0.00025 g/mL from our XG solutions. Compared to XG concentrations used in this study, we also estimated the value of C* using the hydrodynamic coil radius (~110 nm) and found a value of 0.00026 g/mL. We also determined that XG in water at 20 °C produced a shape factor (Equation (12)) value of 2.5 ± 0.1 and indicated a spherical conformation at these conditions. Herein lies one difficulty in relying on viscosity alone to determine conformation. While the power law suggests a semi-rigid rod or coil structure, the shape factor seems to indicate a spherical conformation. So, depending on which parameter(s) is(are) used, one may come to differing perspectives on structural conformation. This underscores the need for further measurements using a technique such as scattering to confidently assign an XG conformation.

### 2.2. Xanthan Gum in Water: Temperature Effects

Having established parameters at 20 °C, we investigated the XG aqueous solution response to temperature variations. The *η*_rel_ decreased by 3% in water as the temperature increased from 20 to 50 °C, whereas at 50 °C, [*η*] increased by 30% compared to 20 °C, suggesting that temperature played a significant role in the XG solution behavior. This behavior has been also reported by Brunchi and co-workers, where they observed that increasing the temperature from 25 to 75 °C resulted in an increased [*η*]~57% [18]. Khouryieh et al. observed a more modest increase in [*η*] of ~12% when varying the temperature from 25 to 80 °C [14]. From [*η*], and hence *η*_sp_, we assessed the XG conformation using Equation (7) by estimating the exponent *b* from the log(*η*_sp_) vs. log([XG]) slope, Figure 2, panel D. Khouryieh et al. reported XG (<MW> = 2.65 × 10^6^ g/mol) slopes of 1.38 and 1.32 for aqueous solution at 25 and 80 °C, respectively [14], whereas our slopes varied from 1.01 to 0.92. Although both data sets report results for XG, the slope variations are attributed to different XG sources and preparation schemes, but nonetheless our data sets still yield similar results.

**Figure 3:**
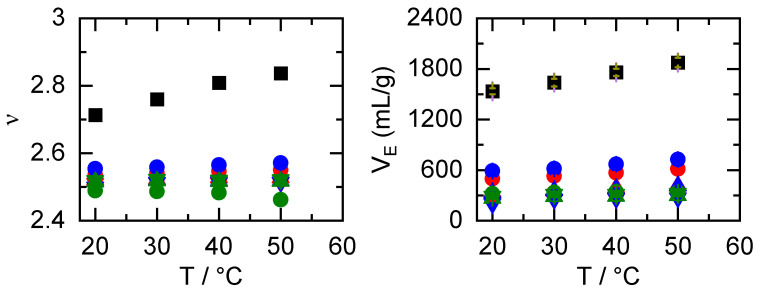
Left panel: shape factor (unitless) for XG in water and salt solutions. Right panel: voluminosity (mL/g) for XG in water and salt solutions. XG in water is presented as square symbols (■). Salt solution data are shown in colored symbols as NaCl (red symbols), KCl (blue symbols), and CaCl_2_ (green symbols). Salt concentrations used are presented as follows: circles for 0.9 mM NaCl (●) and KCl (●), and 0.05 mM CaCl_2_ (●); triangles for 3.0 mM NaCl (▲) and KCl (▲), and 0.4 mM CaCl_2_; (▲) and 100 mM NaCl (▼)and KCl (▼), and 1.14 mM CaCl_2_ (▼). Error bars for these data are from relative standard deviations of replicates and within the symbols’ size, as can be seen in the squares for water.

Further, we computed the voluminosity (*V*_E_) and shape factor (*ν*) to characterize the XG shape in plain water, Figure 3. A decreased *V*_E_ indicates a reduction in the solvation of the XG molecules. *V*_E_ was 22% higher at 50 °C compared to 20 °C and indicated that solvent quality improved with increasing temperature, which is common in salt-free polyelectrolyte solutions. Shape factor was also computed, and our data shows a constant value of 2.5 at all temperatures, consistent with the literature reports and further supporting a spherical conformation [12,27,28]. The XG hydrodynamic coil radius showed linear dependence and increased by 9% over our temperature range. With the lack of literature precedent for XG coil radii behavior, we used nonionic dextran (T2000, 2 × 10^6^ g/mol) as a comparator. While the dextran coil radius decreased by 4% with an increased temperature from 20 °C to 40 °C, in contrast, the XG radius increased by 7% [29]. The dextran structure appears to shrink with temperature whereas XG expands. Likely, the nonionic/anionic differences are the motifs that account for these opposite behaviors. In addition, XG has a smaller gain in solution entropy from its counterion interactions with water, which may also account for the larger XG coil [30]. At higher XG concentrations, chains intermingle by gaining entropy of dissociated counterions, changing the nature of the Coulombic repulsion between charged groups [31]. As the temperature rises to 40 °C, the zero-shear viscosity change seems to be suggestive that the conformation is transforming into one resembling a disordered conformation, resulting in a greater degree of XG flexibility compared to dextran.

### 2.3. Salt Concentration Effect

**Figure 4:**
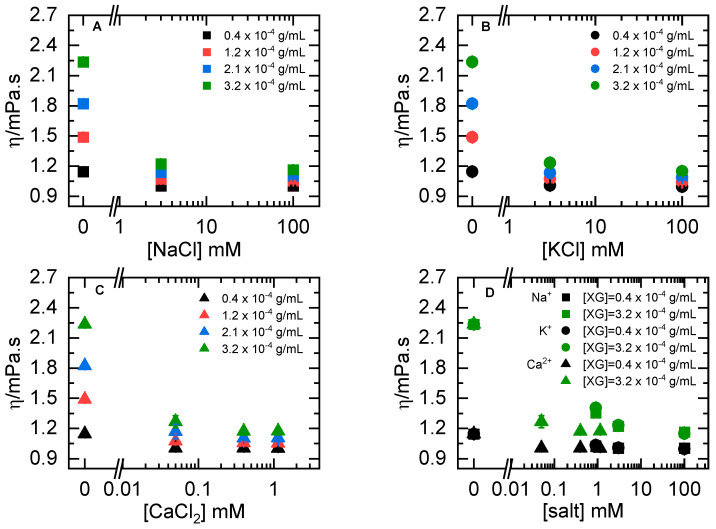
Zero-shear viscosities for varying salt concentrations at 20 °C for four XG concentrations. Panel (**A**) is NaCl data, (**B**) is KCl data, and (**C**) is CaCl_2_ data. For comparative context, panel (**D**) provides an overlay of the specific salt effects.

Having examined the impact of temperature on XG in water, we sought to assess the effect of ionic strength through salt addition. Our interests here were to consider which parameter may be more impactful to XG solution behavior—temperature or salt or a synergistic effect of both. It is also of relevance to practical applications to analyze the influence of salt-containing solutions on the structure of XG. For example, XG finds many applications in various industries such as food, pharmaceutical, and oil and gas extraction where salt concentration and temperature vary [15,16,17]. Thus, studying how these factors affect XG’s performance can help optimize product formulation and process design. This is important to ensure XG’s effectiveness in different system conditions. Given that XG is an anionic polyelectrolyte, the presence of ions in solution are expected to influence structural organization by screening the charged side-chain moieties. This screening effect should result in the formation of a compacted XG structure and be manifested by a decreased measured zero-shear viscosity in the presence of salt compared to water. Figure 4 presents our dynamic viscosity data for varying [XG] as a function of salt concentration, by cation type in Figure 4A–C.

**Figure 5:**
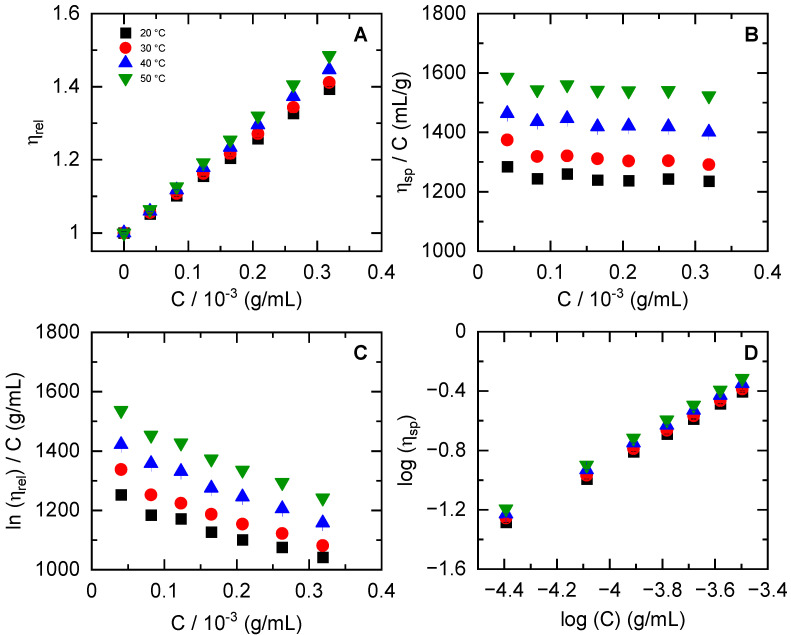
XG viscosity concentration dependence in 0.9 mM NaCl for: (**A**) relative viscosity (*η*_rel_, Equation (4)); (**B**) reduced viscosity (*η*_sp_/C, Equation (3)); (**C**) inherent viscosity (ln(*η*_rel_)/*C*, Equation (2)); and (**D**) specific viscosity (log(*η*_sp_), Equation (7)). In all panels, black symbols (■) are 20 °C, red symbols (●) 30 °C, blue symbols (▲) = 40 °C, and green symbols (▼) = 50 °C. The error bars are relative standard deviations and smaller than the symbols.

Computed representations in Figure 5 for viscosity data (and their magnitudes) depend on solution characteristics such as XG concentration, temperature, ion identity, etc. For example, the relative viscosity change for XG in water was approximately 50% larger, or more, than solutions with added NaCl (see panel A in Figure 2, Figure 5, Figures S1 and S2).

**Figure 6:**
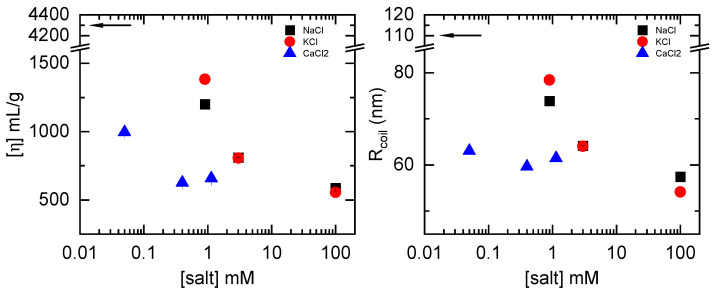
Intrinsic viscosity (**left panel**) and hydrodynamic coil radius (**right panel**) of XG in aqueous solutions of NaCl (■), KCl (●), and CaCl_2_ (▲) at 20 °C. Arrows indicate the values at zero salt concentration. Error bars are from relative standard deviations and typically smaller than the symbols.

Considering a comparable ionic strength to 0.9 mM Na^+^, *η*_rel_ for 0.4 mM divalent Ca^2+^ showed similar overall behavior with [XG]. Figures S3–S5 summarize all *η*_rel_ data for 0.05, 0.4, and 1.14 mM Ca^2+^ solutions. However, the *η*_rel_ value for Ca^2+^ shows smaller values at the same [XG], indicating that the greater charge density of Ca^2+^ is more effective at screening side-chain interactions. For example, at 0.0002 g/mL XG, the *η*_rel_ for Ca^2+^ is 10% smaller than in Na^+^, which, over the concentration range studied, reaches as much as a 17% difference. Temperature has a lessened effect on this salt effect, and within the temperature range 20–50 °C reported here, there is a nominal difference of at most 2% between 20 °C and 50 °C (see Figure 5A and Figure S4). The same general pattern is seen in *η*_sp_/*C*, so which parameters are of more importance depends on the chosen viscosity representation. The reduced viscosity in Figure 5 shows that [XG] variation has a minimal effect because, once the XG structure is compacted by Na^+^, there is apparently little change in XG/solvent interaction. However, a temperature change increased *η*_sp_/C somewhat because of the entropic gain from a thermally expanded structure. On examining [*η*] at 20 °C as shown in Figure 6, left panel, we observed a consistent decrease with added salt relative to water, initially 70% in 0.9 mM NaCl and up to 80% in 100 mM NaCl. Details are summarized in Tables S1–S3 for all salts studied here.

Literature-reported decreases of ~30–45% are in reasonable agreement with our results, given differences such as XG sources, [XG], solution pH, and [Na^+^] (~52.7, 105.4, and 158.2 mM NaCl, three times our values) at 25 °C [11]. The [*η*] reported by Banerjee et al. was 60% smaller than what we observed, but their salt was 1000 mM CaCl_2_·2H_2_O and at 35 °C [12]. The decrease in [*η*] is attributed to the salt ions shielding the XG inter- and intramolecular ionic chain charges. The salt ion screening effect allows XG to assume a more compact solution structure, and, as the chain domains diminish in size, there is an associated reduction in the number of interactions with neighboring chains. In other words, a greater quantity of polymer chains is necessary for the solution to attain an equivalent level of intermolecular interaction and subsequent zero-shear viscosity increase. Consequently, this leads to the reduction in the XG hydrodynamic coil size. We also examined the impact of KCl, as we expected a variation in zero-shear viscosity since the effective ionic radius and affinity differs from NaCl. Figure 6 makes clear the effect of the larger K^+^ cation, which shows that the [*η*] was 20% higher in 0.9 mM K^+^ at 20 °C and was the same in 3 mM solutions to within uncertainty. However, [*η*] was 17% smaller in 100 mM K^+^ than in Na^+^. These results reveal two crucial aspects: (1) XG electrostatic repulsive forces between charged groups are more effective in 100 mM NaCl than KCl; and (2) the larger K^+^ radius (0.138 nm, [32]) and affinity for XG had a greater effect compared to Na^+^ (0.102 nm, [32]) at 20 °C, and thus K^+^ led to a more compact XG conformation [9].

Hydrodynamic coil radius was computed for XG in these salts and over the concentrations studied here and mirrored the observations from [*η*], as is consistent with Equation (8). Figure 6, right panel, shows these changes and gives a comparative perspective of the ion effects. Our data followed the anticipated behavior for polyelectrolytes [33], where the hydrodynamic coil radius decreases with increasing [salt]. We computed and compared the coil radius of the Na^+^, K^+^, and Ca^2+^ and found that the coil radius was ~10% higher in 0.9 mM K^+^ compared to Na^+^ solution, showed no difference for 3 mM salt, and was ~7% smaller at 100 mM, which emphasized the radius differences between Na^+^ and K^+^. Overall, Ca^2+^ was much more effective than the monovalent cations in screening charge, as already described. A coil radius reduction of ~45% was observed for Na^+^ and K^+^ at 20 °C compared to water, showing that initial amounts of added salt have the greatest impact on structural collapse. One point of note, Southwick et al. suggested that at [salt] > ~200 mM, anomalous behavior is observed and the Stokes–Einstein hydrodynamic radius actually increases [33]. We do not observe any indications of anomalous behavior, but our largest concentration is only 100 mM.

As with plain water, the *V*_E_ values in salt solutions were evaluated. The *V*_E_ value for K^+^ was larger than Na^+^ at 0.9 mM salt and comparatively presented a more favorable solvent environment, whereas the reverse is true at 100 mM salt. For Ca^2+^ solutions at 0.4 mM, when considering the estimated monovalent ionic strength equivalent, the divalent cation presented a 50% decreased *V*_E_ value (Figure 3). This decrease indicated that Ca^2+^ created a poorer solvent environment for XG, but this is consistent with the other metrics that showed the divalent ion screening is more significant. The shape factor values were constant for all salt concentrations and indicated that the XG conformation was spherical in the presence of salt.

### 2.4. Temperature Effects: Xanthan Gum in Salts

Reports in the XG literature have suggested that temperature can induce a structural transition from a helical structure to one where XG adopts a more flexible, disordered conformation that leads to an increase in solution viscosity [13,32,34,35]. Intrinsic viscosity is a convenient representation that can be used to track this type of transition. We have used [*η*] to examine the temperature dependence of viscosity, and these results are presented in Figure S6 and Tables S1–S3 for water and salt solutions. From these data, we observed that in general [*η*] increased with increasing temperature, both in the absence and presence of salts, but decreased with [salt]. For example, in 0.9 and 3 mM monovalent salts, [*η*] increased by ~25% from 20 to 50 °C but by only 14% in 100 mM NaCl. For divalent Ca^2+^, [*η*] increased by not more than ~13% over the same temperature range for all [Ca^2+^]. The increased [*η*] indicated a transition from a more compact structure to one of greater chain flexibility. Importantly, the entropic gain through increasing temperature has a greater impact on XG configuration when using monovalent salts to initially compact the structure. Further, cation size also contributed significantly to viscosity change, and we observed that 0.9 mM K^+^ at 50 °C increased [*η*] by 20% over Na^+^, but for 3 mM Na^+^ and K^+^, [*η*] values were similar. In 100 mM monovalent ion solutions, the [*η*] showed a small decrease, by ~5%, likely because, at this higher concentration, the side chains are mostly “saturated” with salt and the effect is diminished. Then, for similar ionic strengths of Na^+^ or K^+^ with Ca^2+^ at 50 °C, [*η*] was smaller by ~50% and 60%, respectively, in the divalent salt. This diminished [*η*] also supports the idea of less chain interaction with the divalent cation, coupled to a decrease in Coulombic interactions because of charge screening. Complimentary temperature-dependent salt solution data for [*η*] are in Figure S6 and those for coil radii are in Figure S7. The trends are in parallel to what was described for [*η*] values.

We conducted a comparison to better understand how salts and temperature affect the solution structure of XG. Specifically, we explored how they modulate the solvent quality of XG macromolecules using the swollen specific volume or *V*_E_. When assessing the interaction between a solvent and a macromolecule, *V*_E_ considers the macromolecule volume in a solution relative to its anhydrous mass [36]. We observed that *V*_E_ increased by 11–12% in 0.9 and 3 mM, Na^+^, and K^+^, from 20 to 50 °C. For the same temperature range in a 100 mM Na^+^ solution, the *V*_E_ only rose by 8%, but for K^+^, the increase was 25%. These results demonstrate that the solvent quality was affected by not only ionic strength but also ion size. The reduction in *V*_E_ is also linked to the salt screen effect, which led to a more compact configuration. Another possible contribution might be related to gelling formation, for which we suggest further investigations are needed for confirmation of the effect. With the introduction of the divalent Ca^2+^ ion, *V*_E_ experienced an overall 7% increase as the temperature was raised to 50 °C. Comparing the similar ionic strength Na^+^ and K^+^ salt solutions at 50 °C, *V*_E_ increased by ~18% in 0.9 mM K^+^ compared to Na^+^ solution. *V*_E_ was smaller by approximately 49% and 56% in the presence of divalent ions for solutions with similar ionic strength of Na^+^ or K^+^ with Ca^2+^. These data concur with intrinsic viscosity results. Amini et al. also observed a similar decrease (~45%) in solvent quality in 0.05 mM Ca^2+^ with Balangu seed gum solution compared to Na^+^ [37]. In addition, Yousefi et al. showed that the *V*_E_ values of sage seed gum were more strongly affected by Ca^2+^ than Na^+^ [38]. This demonstrates that Ca^2+^ has a more significant impact on XG solvation, resulting in less extended chain conformation. The general observation is that, as the ionic strength increases, XG structures tend to become more compact in configuration, as evidenced by the reduction of *V*_E_. The reduction in molecular dimension is a result of the charge screen effect on the electrostatic repulsions of the tri-saccharide side chains, leading to a more compact conformation [21]. This causes a decrease in the hydrodynamic coil radius of XG in solution. Further, it is important to point out that the solvent quality can be also affected by the ionic nature of XG macromolecules in solution, since XG is a polyelectrolyte. This means that XG properties are manifestations of the electrostatic interactions between ionized charged groups. However, it appears that the solvent quality is more affected by changes in ionic strength than by changes in temperature. The shielding effect of electrostatic interactions that occurs at higher salt concentrations is controlled by the salt ions and the electrostatic interactions between charged ionized groups on the XG side chain. As the screening effect increased, the physical properties of XG solutions are similar to those in a solution of neutral polymer in a good solvent [39].

### 2.5. Model of [η]—Huggins and Kraemer Constants

The accurate determination of a polymer’s size and conformation in solution is of primary importance, because the contribution of structure to application will depend on its molecular organization. Although viscosity does not give direct structural information, significant insight can be gained from [*η*]. Intrinsic viscosity plays a crucial role in this regard because [*η*] is a good measure of a polymer’s ability to increase solvent viscosity [40]. Given the numerous models proposed and still under development for quantifying [*η*], it is imperative to conduct cross-checks and comparisons of these models when assessing the solution structure of the polymer in dilute solutions [41,42,43,44,45,46]. This is particularly significant for polyelectrolytes, as their solution structure is considerably influenced by several factors, including polymer concentration, pH, salt concentration, salt identity, and temperature. Thus, careful evaluation and analysis using various models can furnish additional information for polyelectrolytes’ characterization. For this reason, [*η*] values of XG in water and salt solutions were calculated using five models (Equations (2)–(6)), and the results are summarized in Tables S1–S3. Values estimated using the Rao 1 model in water, with varied temperatures, were closer to those obtained using the Huggins and Kraemer equations. However, [*η*] calculated using Rao 2 and Rao 3 yielded smaller values in the absence of salt. Work by the Bak and Higiro groups reported similar observations and suggested that, of these models, Rao 1 supplied the best agreement for intrinsic viscosity and that Rao 2 and Rao 3 did not give acceptable results for XG in water solutions [10,11]. However, no quantitative assessment/comparison was reported for the variation in model performance. In our work with water, the variation among models is between 140–200% over the temperature range 20–50 °C. When salt was introduced into XG solutions, the differential between models became smaller as the salt concentration was increased, such that in 100 mM monovalent salt and 1.14 mM divalent salt, all models gave reasonably similar results. Comparatively, for our data at the smallest salt concentrations, the model variation was between 35–50%, whereas, at the highest salt concentrations, the variation is only 20–25%. Clearly, there is better agreement among models at the larger salt concentrations and higher temperatures where charge is screened and XG structure is expanded. The result is that XG in the presence of salt appears to behave like a neutral polymer.

In this study, we assumed that the Huggins and Kraemer equations are applicable to weak polyelectrolyte solution with varied salt concentrations. The Huggins equation is usually applied to neutral polymers, and with weakly acidic (-COOH) groups, XG appears to be in this category of polyelectrolytes [12]. The Huggins (*K*_H_) and Kraemer (*K*_K_) constants are used as parameters for characterizing the solvent quality. We have calculated the *K*_H_ and *K*_K_ coefficients, which are reported in Tables S1–S3. Our results showed variation between positive and negative values of *K*_H_ constants, which typically are positive, and negative values of *K*_K_ constants that are typically negative. The *K*_H_ values are often not reported in the XG literature, because the plot of the Huggins equation decreases with increasing polyelectrolyte concentrations, leading to a negative Huggins constant. These negative values then correspond to polymer immiscibility [47]. For a polymer in a suitable solvent, the *K*_H_ value should lie between 0.3 and 0.8, and higher values than 1.0 suggest a polymer–polymer aggregation [29]. The sum of *K*_H_ and *K*_K_ coefficients should equal 0.5 (theoretical) for a polymer in a suitable solvent, while values greater than or less than 0.5 indicates molecular association [10]. Variation in *K*_H_ points to solvent quality alteration with the variation in salt concentration, salt identity, and temperature. For example, in water solutions, reduced viscosity decreased with increased XG concentrations, resulting in a negative *K*_H_, and so indicated a polyelectrolyte behavior. However, in 100 mM NaCl solution, the *K*_H_ value was ≥0.2 and therefore suggested that, as Na^+^ screened the charged groups on the XG backbone chain, XG behaved like a neutral polymer. In contrast, the negative *K*_H_ values for K^+^ and Ca^2+^ seem to suggest that both ion size and charge density are important points of consideration in controlling the ultimate XG properties. These results may be interpreted to show structural (re-)organization of XG in K^+^ and Ca^2+^ and illuminate in what ways it may be different than in Na^+^. However, one should not rely only on the Huggins constant to explain the XG behavior in aqueous salt solutions. In addition, it may be necessary to investigate the formation of ionic crosslinking and the role that K^+^ and Ca^2+^ have on the XG structure. These salts have the potential to impact both the XG network structure and solution viscosity. Here, we suggest that further structural studies are required to more thoroughly investigate how Na^+^, K^+^, Ca^2+^, and other cations influence the solution structure.

Our experimental observations of the *K*_H_ coefficient can be summarized as follows: (a) *K*_H_ is more sensitive to salt identity than temperature; (b) *K*_H_ is sensitive to conformation transition, network formation, or molecular aggregation [10]; and (c) when XG–XG interactions become more favorable than XG–solvent interactions, the *K*_H_ value can be indicative of the behavior. For example, assuming a flexible polymer *K*_H_ is expected to be greater than 0.5 for polymers in poor solvents [48], it is greater than 1.0 if the polymer behavior mimics uncharged solid spheres [12] or it is less than 0.1 to even negative values, which may suggest immiscibility or gelation. Furthermore, as the *K*_K_ coefficient decreases towards zero, indicating a poor solvent, and subsequently becomes positive while continuing to increase, it suggests a decline in the thermodynamic quality of the solvent [49]. The behavior was noticed when K^+^ and Ca^2+^ were present in XG solutions and suggested that the sign change from negative to positive indicated XG–XG interactions rather than XG–solvent interactions. The *K*_K_ coefficients can provide additional insights into the manifestation of XG–XG interactions in a solution. Importantly, establishing the *K*_H_ and *K*_K_ for XG under different conditions is crucial, since these parameters are scarce in the literature.

## 3. Materials and Methods

### 3.1. Materials and Sample Preparation

Xanthan gum used in this study is commercially available food grade from CPKelco, Atlanta, GA, material number: 10040281. We contacted CPKelco to inquire about the XG manufacturing process used and the CPKelco response stated, “After most of the isopropyl alcohol is removed, the xanthan gum fibers are subjected to drying in rotary dryers. The critical drying parameters include temperature and relative humidity. The acceptable ranges for these critical parameters are considered confidential”. The polymer was received in powder form and used without further purification or modification. A 0.5 wt% XG stock solution was prepared using deionized water (18.2 MΩ cm) from a MilliQ system. The solution was gently stirred overnight to obtain a hydrated and equilibrated solution that appeared visually transparent, suggesting solution homogeneity with respect to the issue of aggregate formation. CPKelco’s Product Data Sheet and Guidelines for Proper Dissolution says, “A good dispersion rate is one that allows for slow-enough addition to ensure no lump formation. KELTROL xanthan gum will hydrate in most water-based systems because it is completely soluble in both hot and cold water. Solutions are usually made by sifting dry KELTROL into water with sufficient agitation to bring about a physical separation of the particles”. The solution was kept refrigerated at 4 °C to minimize bacterial growth but was equilibrated at the desired experimental temperature for not less than 60 min before any measurement. NaCl, KCl, and CaCl_2_·2H_2_O were purchased from Fisher Scientific (Hampton, NH, USA) and used as received. Solutions for measurement were prepared by diluting a 0.5 wt% XG stock solution to the desired concentrations and equilibrated at 20 °C for at least 30 min prior to measuring. [Na^+^] and [K^+^] were 0.9 mM, 3 mM, and 100 mM, similar to literature [9]. [Ca^2+^] were 0.05, 0.40, and 1.14 mM, as CaCl_2_·2H_2_O.

All data reported here were based on average of at least three replicate measurements, followed by the propagation of measurement uncertainties from all raw measurement parameters.

### 3.2. Instrumentation and Measurements

Density and viscosity of aqueous xanthan gum at each solution concentration were simultaneously measured with an Anton-Paar (Anton-Paar-Str. 20, A-8054, Graz, Austria) integrated density/viscosity instrument, consisting of the DMA4100 density meter and the Lovis 2000 ME rolling ball viscometer outfitted with a quartz capillary (Mat. No. 73109, 1.59 mm) and a 1.5 mm diameter and 7.67 g/cm^3^ steel ball. The ball rolls through the thermostated capillary and the instrument measures the time required for the ball to travel between two fixed points within the sensor housing. From the timing, sample dynamic viscosity is computed from the following equation that is given in the instrument’s manual:

*η* = *K* Δ*t* (*ρ*_b_ − *ρ*_s_), (1)

where *η* is the measured sample viscosity, *K* is the capillary calibration constant, Δ*t* is the rolling runtime, and *ρ*_b_ and *ρ*_s_ are the densities of the ball and the sample, respectively. The constant *K* must be determined using a known viscosity calibration standard. For our work, we calibrated the instrument at the desired working temperatures using a certified degassed ultrapure water sample from Anton Paar that remained sealed in a crimped vial until use. The rolling time Δ*t* and *K*, and thus the dynamic viscosity, depend on the angle of inclination, *θ*. Sample shear results from the ball rolling at the capillary angle *θ*. Since each single measurement is influenced by the mean shear rate, the manufacturer’s recommended procedure is to measure samples using a set of different *θ* values to determine the zero-shear viscosity. For our work, inclination angles falling in the range of 20–70° with respect to 0° (the instrument’s horizontal position) were selected automatically by the instrument software according to the rolling time. Relative viscosity (*η*_rel_ = *η*(*θ*)/*η*_w_) was plotted versus sin(*θ*), and the data were regressed using a linear model to determine the y-intercept. *η*_w_ is the water viscosity at 293 K (1.002 mPa s) and the intercept is *η*_0_, the zero-shear relative viscosity of the sample. A minimum of six replicates were acquired for each sample at all temperatures measured, and the measured viscosities are a data set average. Viscometer rolling angles were accurate to ±0.1°. Temperatures were varied between 20 and 50 °C, and each was maintained to within ±0.02 K. For all viscosity measurements, the uncertainty is at most ±0.1%, and reproducibility variation was less than ±0.6%.

### 3.3. Analysis

Sample viscosities were used to compute the relative viscosity at zero-shear rate using *η*_rel_ = *η*/*η*_0_, where *η* and *η*_0_ are the solution and pure solvent viscosities, respectively, and the specific viscosity was computed as *η*_sp_ = *η* − 1. Intrinsic viscosity, [*η*], was calculated using the Huggins and Kraemer models, Equations (2) and (3) [29]:

ln(*η*_rel_)/*C* = [*η*] + *K*_K_[*η*]^2^*C*, (2)

*η*_sp_/*C* = [*η*] + *K*_H_[*η*]^2^*C*, (3)

where *C* is polymer concentration (g/mL), ln(*η*_rel_)/*C* is inherent viscosity, *η*_sp_/*C* is reduced viscosity, *K*_K_ is the Kraemer constant, *K*_H_ is the Huggins constant, and *η*_sp_ is specific viscosity. The Kraemer (*K*_K_) and Huggins (*K*_H_) constants are extracted from the slopes in Equations (2) and (3). The intercept from extrapolation to zero polymer concentration gives the [*η*]. For polyelectrolytes in salt-free aqueous solution, the reduced viscosity should decrease with an increasing polymer concentration. Estimating [*η*] from the intercept using the Huggins model limits its reliability. However, [*η*] can be reliably estimated from *η*_rel_ as a function of xanthan gum concentration. In addition, three models proposed by Tanglertpaibul and Rao have been used to determine [*η*], and were compared with the Huggins and Kraemer equations [14]. The Rao forms are [46]:

*η*_rel_ = 1 + [*η*]*C*, (4)

*η*_rel_ = exp ([*η*]*C*), (5)

(6) $$\eta_{rel}=\frac{1}{1-\left(\left[\eta \right]C\right)}$$

These models are suggested as the best models to estimate [*η*] polyelectrolyte aqueous solution [10,11,14,45,46]. We computed [*η*] from Equations (4)–(6) and compared it to values obtained from intercepts to assess the effect of salt and temperature on XG solutions. One can also evaluate the XG solution conformation by fitting data to a power-law dependence of the general form:

log(*η*_sp_) = log(*a*) + *b* log(*C*_XG_), (7)

where the value of *b* indicates conformation [10,50]. For *b* < 1, a rod-like conformation is suggested and values for *b* > 1 are ascribed to a coil conformation. The hydrodynamic coil radius of XG chains (in cm) was computed according to [27,51]:

(8) $$R_{coil}={\left[\frac{3\left[\eta \right]MW}{10\pi N_{A}}\right]}^{1/3},$$

where *N*_A_ is Avogadro’s number and *MW* is the XG molecular weight, reported as 2.0 × 10^6^ g/mol by Wyatt and Liberatore, who also acquired XG from CPKelco [52]. The macromolecular overlap critical concentration, C*, marks the crossover point between the dilute and concentrated regimes. C* is estimated from the ideal polymer coil volume using the experimentally determined radius of gyration, *R*_g_, calculated from [*η*] via the Flory–Fox equation [26,53]:

(9) $$R_{g}={\left(\frac{[\eta ]MW}{\Phi_{O}}\right)}^{1/3},$$

then, the overlap concentration, C* follows:

(10) $$C^{*}=\left(\frac{MW/N_{A}}{\frac{4}{3}\pi R_{g}^{3}}\right)=\frac{\Phi_{O}}{\frac{4}{3}\pi N_{A}\left[\eta \right]},$$

where variables and constants are previously defined [53,54,55]. Finally, the swollen specific volume or voluminosity (*V*_E_)

(11) $$Y=\frac{\left(\eta_{rel}^{0.5}-1\right)}{C\left(1.35\;\eta_{rel}^{0.5}-0.1\right)},$$

and shape factor (*ν*)

[*η*] = *ν V*_E_, (12)

are additional parameters used to describe solution characteristics [27,28]. *V*_E_ is obtained from the intercept by plotting Y as a function of XG concentration (g/mL), which gives a measure of the solvated polymer volume and also conveys information regarding the polymer conformation [27]. Macromolecular shape is estimated from *ν*, where a value of *ν* ~ 2.5 suggests a spherical conformation and *ν* > 2.5 suggests ellipsoidal particles [56].

## 4. Conclusions

We investigated the influence of salt on aqueous XG solutions by varying [XG], salt identity, and concentration, along with temperature. Collectively, all our observations consistently suggested that the greater impact to XG solution behavior is from the salt concentration and cation identity. In water, [*η*] increased by 30% from 20 to 50 °C, showing that temperature played an important role in the XG solution organization, which translated to an increased hydrodynamic coil radius by 9%. In the presence of Na^+^ and K^+^ (compared to plain water) at 20 °C, the coil radius decreased by ~45%. This provided a clear indication that salt shielding resulted in a more compact XG structure. When considering similar ionic strength solutions at 50 °C, e.g., 0.4 mM Ca^2+^ and 0.9 mM Na^+^ and K^+^, [*η*] values in the divalent salt solution were smaller than those in the monovalent ion solutions by ~50% and 60%, respectively, and better screened the charged groups on the XG side chains [24]. The power-law slopes were broadly in the range of 1.0 ± 0.1 and reasonably similar across salt identities and temperatures. Since slope values greater than 1.0 are typical for random coil conformation, and values less than 1.0 are more indicative of a rod-like conformation; these XG solutions lie in the middle of these limits, so specific structural implications based on zero-shear viscosity measurements alone are not conclusive. However, the shape factor (*v*) computation for our data was close to 2.5, which suggested these solutions produced a nearly spherically shaped conformation.

While the specific nature of XG conformational change cannot be unequivocally established, the viscosity parameters reported here can be used to infer conformation or conformation change. The physical picture that emerges from all the results and analysis presented here is that salt concentration, salt identity—specifically monovalent, divalent cations—and ion size collectively influence the solution structure of XG more than temperature. We observed that temperature, in fact, works against the salt effect and strengthens XG electrostatic interactions. We have established that, to properly analyze the impact of salt and temperature on XG solution structure, it is crucial to utilize various models in order to comprehend how polymer structural change may affect the solution. We observed that added salt produced a coil radius 50% smaller than in plain water; however, this decrease cannot be considered conclusive evidence of the disorder-to-order transitions discussed in the literature [9,12,14,57]. This work shows the need for studies that can reveal structural information with the aim of unequivocally determining the details of XG structure and conformational change with varying solvent conditions.

## Figures and Tables

**Table 1:** Slopes (*b*) from Equation (7) for XG in water and aqueous salt solutions.

	NaCl			
T/°C	0 mM	0.9 mM	3 mM	100 mM
20	0.982 ± 0.008	0.984 ± 0.005	0.985 ± 0.005	0.996 ± 0.003
30	0.94 ± 0.02	0.974 ± 0.005	0.985 ± 0.004	0.990 ± 0.005
40	0.92 ± 0.03	0.981 ± 0.003	0.976 ± 0.003	0.996 ± 0.004
50	0.91 ± 0.03	0.984 ± 0.004	0.991 ± 0.003	0.99 ± 0.01
	KCl			
20	0.982 ± 0.008	0.95 ± 0.01	0.98 ± 0.02	1.055 ± 0.007
30	0.94 ± 0.02	0.951 ± 0.008	0.96 ± 0.01	0.95 ± 0.01
40	0.92 ± 0.03	0.947 ± 0.009	0.98 ± 0.02	0.95 ± 0.01
50	0.91 ± 0.03	0.94 ± 0.01	0.98 ± 0.02	0.95 ± 0.02
	CaCl_2_·2H_2_O			
	0 mM	0.05 mM	0.4 mM	1.14 mM
20	0.982 ± 0.008	1.11 ± 0.03	0.97 ± 0.01	0.94 ± 0.02
30	0.94 ± 0.02	1.12 ± 0.03	0.96 ± 0.02	0.93 ± 0.02
40	0.92 ± 0.03	1.14 ± 0.02	0.98 ± 0.02	0.95 ± 0.01
50	0.91 ± 0.03	1.18 ± 0.02	0.98 ± 0.01	0.94 ± 0.02

## Data Availability

Data may be made available upon request.

## Supplementary Materials

The following supporting information can be downloaded at: https://www.mdpi.com/article/10.3390/ijms25010490/s1.

## References

[1] BeMiller J.N. (2019). 4.1 Chemical Structures and Names of Polysaccharides. Carbohydrate Chemistry for Food Scientists.

[2] Meng Y., Nicolai T., Benyahia L., Nicol E. (2022). Utilization of xanthan to stabilize water in water emulsions and modulate their viscosity. Carbohydr. Polym..

[3] Nsengiyumva E.M., Alexandridis P. (2022). Xanthan gum in aqueous solutions: Fundamentals and applications. Int. J. Biol. Macromol..

[4] Jansson P.-E., Kenne L., Lindberg B. (1975). Structure of the extracellular polysaccharide from xanthomonas campestris. Carbohydr. Res..

[5] Morris E.R. (2019). Ordered conformation of xanthan in solutions and “weak gels”: Single helix, double helix—Or both?. Food Hydrocoll..

[6] Bercea M., Morariu S. (2020). Real-time monitoring the order-disorder conformational transition of xanthan gum. J. Mol. Liq..

[7] Shibaev A.V., Muravlev D.A., Muravleva A.K., Matveev V.V., Chalykh A.E., Philippova O.E. (2020). pH-Dependent Gelation of a Stiff Anionic Polysaccharide in the Presence of Metal Ions. Polymers.

[8] Brunchi C.-E., Morariu S., Bercea M. (2021). Impact of ethanol addition on the behaviour of xanthan gum in aqueous media. Food Hydrocoll..

[9] Brunchi C.E., Morariu S., Bercea M. (2014). Intrinsic viscosity and conformational parameters of xanthan in aqueous solutions: Salt addition effect. Colloid Surf. B.

[10] Higiro J., Herald T.J., Alavi S. (2006). Rheological study of xanthan and locust bean gum interaction in dilute solution. Food Res. Int..

[11] Bak J.H., Yoo B. (2018). Intrinsic viscosity of binary gum mixtures with xanthan gum and guar gum: Effect of NaCl, sucrose, and pH. Int. J. Biol. Macromol..

[12] Banerjee P., Mukherjee I., Bhattacharya S., Datta S., Moulik S.P., Sarkar D. (2009). Sorption of Water Vapor, Hydration, and Viscosity of Carboxymethylhydroxypropyl Guar, Diutan, and Xanthan Gums, and Their Molecular Association with and without Salts (NaCl, CaCl_2_, HCOOK, CH_3_COONa, (NH_4_)(2)SO_4_ and MgSO_4_) in Aqueous Solution. Langmuir.

[13] Brunchi C.-E., Bercea M., Morariu S., Dascalu M. (2016). Some properties of xanthan gum in aqueous solutions: Effect of temperature and pH. J. Polym. Res..

[14] Khouryieh H.A., Herald T.J., Aramouni F., Alavi S. (2006). Influence of mixing temperature on xanthan conformation and interaction of xanthan–guar gum in dilute aqueous solutions. Food Res. Int..

[15] Habibi H., Khosravi-Darani K. (2017). Effective variables on production and structure of xanthan gum and its food applications: A review. Biocatal. Agric. Biotechnol..

[16] Singhvi G., Hans N., Shiva N., Kumar Dubey S., Hasnain M.S., Nayak A.K. (2019). Xanthan gum in drug delivery applications. Natural Polysaccharides in Drug Delivery and Biomedical Applications.

[17] Wever D.A.Z., Picchioni F., Broekhuis A.A. (2011). Polymers for enhanced oil recovery: A paradigm for structure-property relationship in aqueous solution. Prog. Polym. Sci..

[18] Brunchi C.-E., Avadanei M., Bercea M., Morariu S. (2019). Chain conformation of xanthan in solution as influenced by temperature and salt addition. J. Mol. Liq..

[19] Potier M., Tea L., Benyahia L., Nicolai T., Renou F. (2020). Viscosity of Aqueous Polysaccharide Solutions and Selected Homogeneous Binary Mixtures. Macromolecules.

[20] Mirabolhassani S.E., Rafe A., Razavi S.M.A. (2016). The influence of temperature, sucrose and lactose on dilute solution properties of basil (*Ocimumbasilicum*) seed gum. Int. J. Biol. Macromol..

[21] Yousefi A., Elmarhoum S., Khodabakhshaghdam S., Ako K., Hosseinzadeh G. (2022). Study on the impact of temperature, salts, sugars and pH on dilute solution properties of Lepidium perfoliatum seed gum. Food Sci. Nutr..

[22] Nickerson M.T., Paulson A.T., Hallett F.R. (2004). Dilute solution properties of κ-carrageenan polysaccharides: Effect of potassium and calcium ions on chain conformation. Carbohydr. Polym..

[23] Cai W.D., Qiu W.Y., Ding Z.C., Wu L.X., Yan J.K. (2019). Conformational and rheological properties of a quaternary ammonium salt of curdlan. Food Chem..

[24] Ghasemi M., Tsianou M., Alexandridis P. (2017). Assessment of solvents for cellulose dissolution. Bioresour. Technol..

[25] Behrouzian F., Razavi S.M.A., Karazhiyan H. (2014). Intrinsic viscosity of cress (*Lepidium sativum*) seed gum: Effect of salts and sugars. Food Hydrocoll..

[26] Morris E.R., Cutler A.N., Ross-Murphy S.B., Rees D.A., Price J. (1981). Concentration and shear rate dependence of viscosity in random coil polysaccharide solutions. Carbohydr. Polym..

[27] Antoniou E., Themistou E., Sarkar B., Tsianou M., Alexandridis P. (2010). Structure and dynamics of dextran in binary mixtures of a good and a bad solvent. Colloid Polym. Sci..

[28] Fathi M., Mohebbi M., Koocheki A. (2016). Introducing Prunus cerasus gum exudates: Chemical structure, molecular weight, and rheological properties. Food Hydrocoll..

[29] Antoniou E., Tsianou M. (2012). Solution properties of dextran in water and in formamide. J. Appl. Polym. Sci..

[30] Kronberg B., Holmberg K., Lindman B. (2014). Surface Chemistry of Surfactants and Polymers.

[31] Muthukumar M. (2017). 50th Anniversary Perspective: A Perspective on Polyelectrolyte Solutions. Macromolecules.

[32] Valente E.C., Poleto M.D., de Oliveira T.V., Soares L.d.S., dos Reis Coimbra J.S., Guimaraes A.P., de Oliveira E.B. (2022). Effects of the Cations Li^+^, Na^+^, K^+^, Mg^2+^, or Ca^2+^ on Physicochemical Properties of Xanthan Gum in Aqueous Medium—A view from Computational Molecular Dynamics Calculations. Food Biophys..

[33] Southwick J.G., Jamieson A.M., Blackwell J. (1982). Conformation of xanthan dissolved in aqueous urea and sodium chloride solutions. Carbohydr. Polym..

[34] Nsengiyumva E.M., Heitz M.P., Alexandridis P. (2023). Thermal hysteresis phenomena in aqueous xanthan gum solutions. Food Hydrocoll..

[35] Morris E.R., Rees D.A., Young G., Walkinshaw M.D., Darke A. (1977). Order-disorder transition for a bacterial polysaccharide in solution. A role for polysaccharide conformation in recognition between Xanthomonas pathogen and its plant host. J. Mol. Biol..

[36] Hesarinejad M.A., Razavi S.M.A., Koocheki A. (2015). Alyssum homolocarpum seed gum: Dilute solution and some physicochemical properties. Int. J. Biol. Macromol..

[37] Mohammad Amini A., Razavi S.M.A. (2012). Dilute solution properties of Balangu (*Lallemantia royleana*) seed gum: Effect of temperature, salt, and sugar. Int. J. Biol. Macromol..

[38] Yousefi A.R., Razavi S.M., Khodabakhsh Aghdam S.H. (2014). Influence of temperature, mono- and divalent cations on dilute solution properties of sage seed gum. Int. J. Biol. Macromol..

[39] Dobrynin A.V., Jacobs M. (2021). When Do Polyelectrolytes Entangle?. Macromolecules.

[40] Lu Y., An L., Wang Z.-G. (2013). Intrinsic Viscosity of Polymers: General Theory Based on a Partially Permeable Sphere Model. Macromolecules.

[41] Rutgers I.R. (1962). Relative viscosity and concentration. Rheol. Acta.

[42] Yan D. (2015). Intrinsic viscosity of polymer solutions: Fresh ideas to an old problem. Sci. China Chem..

[43] Mead D.J., Fuoss R.M. (1942). Viscosities of Solutions of Polyvinyl Chloride. J. Am. Chem. Soc..

[44] Huggins M.L. (1942). The viscosity of dilute solutions of long-chain molecules. IV. Dependence on concentration. J. Am. Chem. Soc..

[45] Rao M.V.S. (1993). Viscosity of dilute to moderately concentrated polymer solutions. Polymer.

[46] Tanglertpaibul T., Rao M.A. (1987). Intrinsic Viscosity of Tomato Serum as Affected by Methods of Determination and Methods of Processing Concentrates. J. Food Sci..

[47] Chee K.K. (1990). Determination of polymer-polymer miscibility by viscometry. Eur. Polym. J..

[48] Ma X., Pawlik M. (2007). Intrinsic viscosities and Huggins constants of guar gum in alkali metal chloride solutions. Carbohydr. Polym..

[49] Gosteva A., Gubarev A.S., Dommes O., Okatova O., Pavlov G.M. (2023). New Facet in Viscometry of Charged Associating Polymer Systems in Dilute Solutions. Polymers.

[50] Chronakis I.S., Alexandridis P. (2001). Rheological Properties of Oppositely Charged Polyelectrolyte−Surfactant Mixtures:  Effect of Polymer Molecular Weight and Surfactant Architecture. Macromolecules.

[51] Antoniou E., Alexandridis P. (2010). Polymer conformation in mixed aqueous-polar organic solvents. Eur. Polym. J..

[52] Wyatt N.B., Liberatore M.W. (2010). The effect of counterion size and valency on the increase in viscosity in polyelectrolyte solutions. Soft Matter.

[53] Vadillo D.C., Mathues W., Clasen C. (2012). Microsecond relaxation processes in shear and extensional flows of weakly elastic polymer solutions. Rheol. Acta.

[54] Rodrigues T., Galindo-Rosales F.J., Campo-Deaño L. (2020). Critical overlap concentration and intrinsic viscosity data of xanthan gum aqueous solutions in dimethyl sulfoxide. Data Brief.

[55] Graessley W.W. (1980). Polymer chain dimensions and the dependence of viscoelastic properties on concentration, molecular weight and solvent power. Polymer.

[56] Rangaraj A., Vangani V., Rakshit A.K. (1997). Synthesis and characterization of some water soluble polymers. J. Appl. Polym. Sci..

[57] Rochefort W.E., Middleman S. (1987). Rheology of Xanthan Gum: Salt, Temperature, and Strain Effects in Oscillatory and Steady Shear Experiments. J. Rheol..

