# Supplementary material for: Salt and Temperature Effects on Xanthan Gum Polysaccharide in Aqueous Solutions
Source: Int J Mol Sci. 2023 Dec 29;25(1):490. doi: 10.3390/ijms25010490 (PMC10778890; doi:10.3390/ijms25010490)
Supplement: Supplementary file 1 [file ijms-25-00490-s001.zip › ijms-2719221-supplementary.pdf]

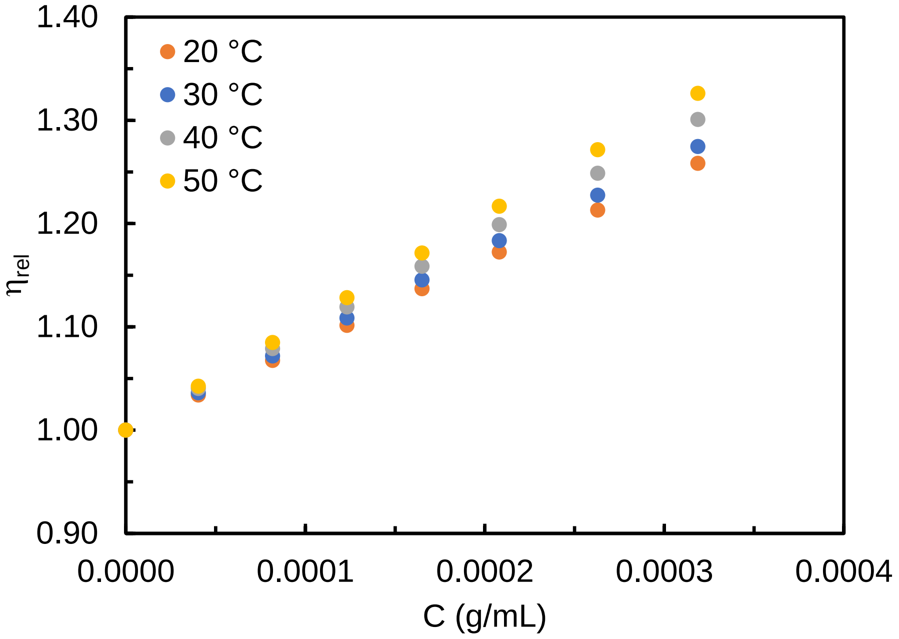

Figure S1. Xanthan gum relative viscosity as a function of xanthan gum concentration in 3 mM NaCl at varied temperatures.

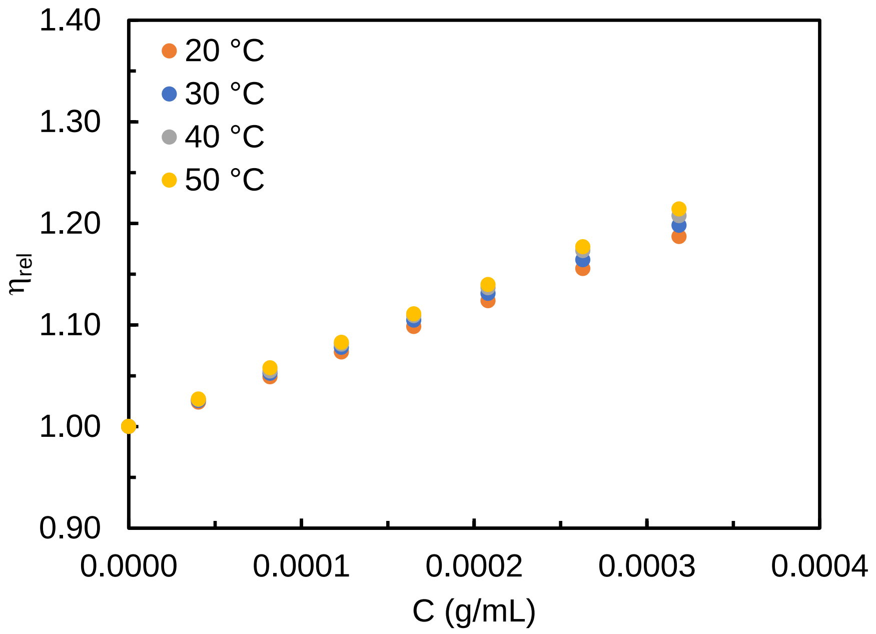

Figure S2. Xanthan gum relative viscosity as a function of xanthan gum concentration in 100 mM NaCl at varied temperatures.

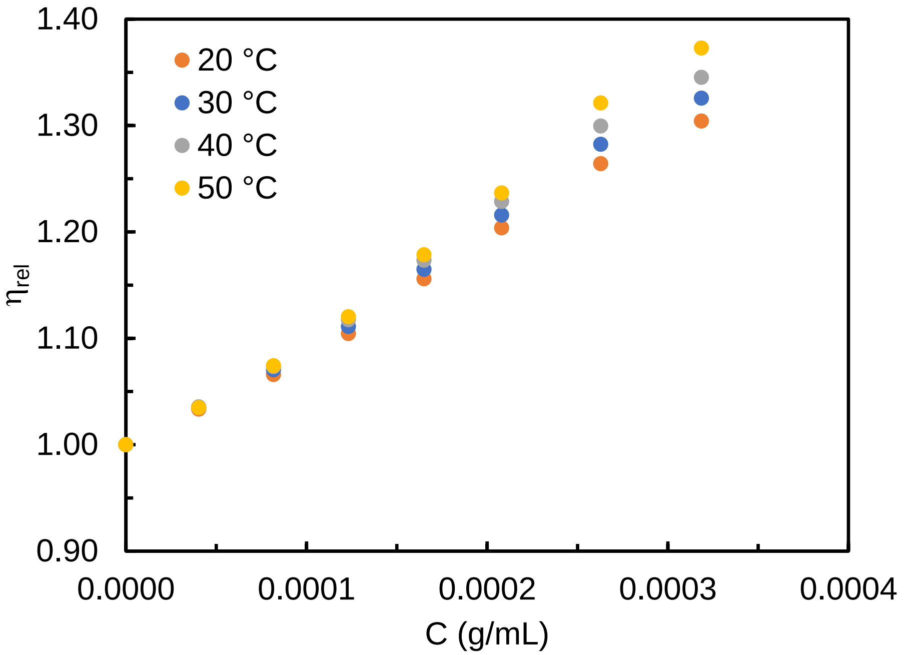

Figure S3. Xanthan gum relative viscosity as a function of xanthan gum concentration in 0.05 mM $CaCl_2{\times}2H_2O$ at varied temperatures.

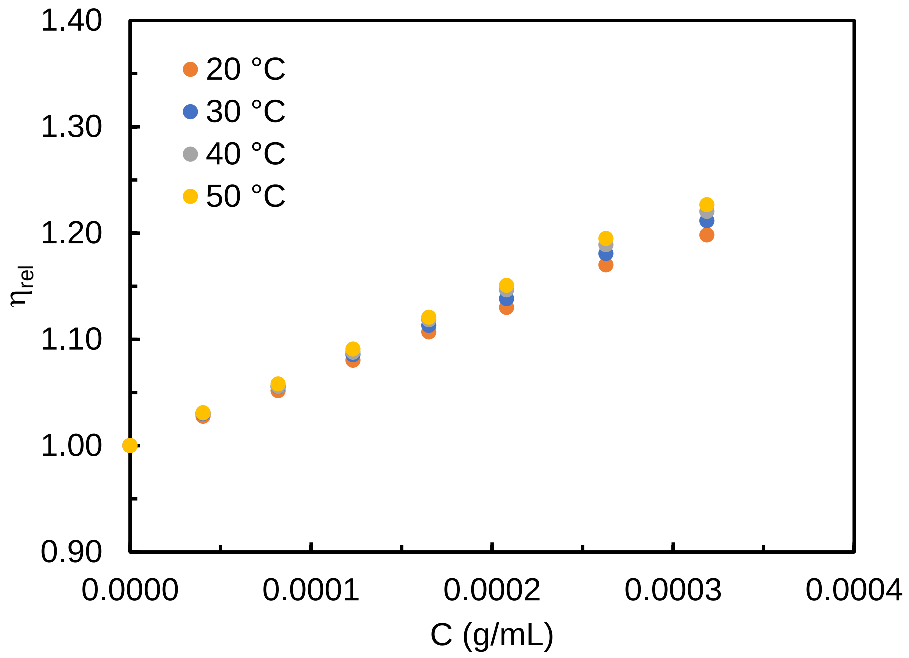

Figure S4. Xanthan gum relative viscosity as a function of xanthan gum concentration in 0.4 mM $CaCl_2 \times 2H_2O$ at varied temperatures.

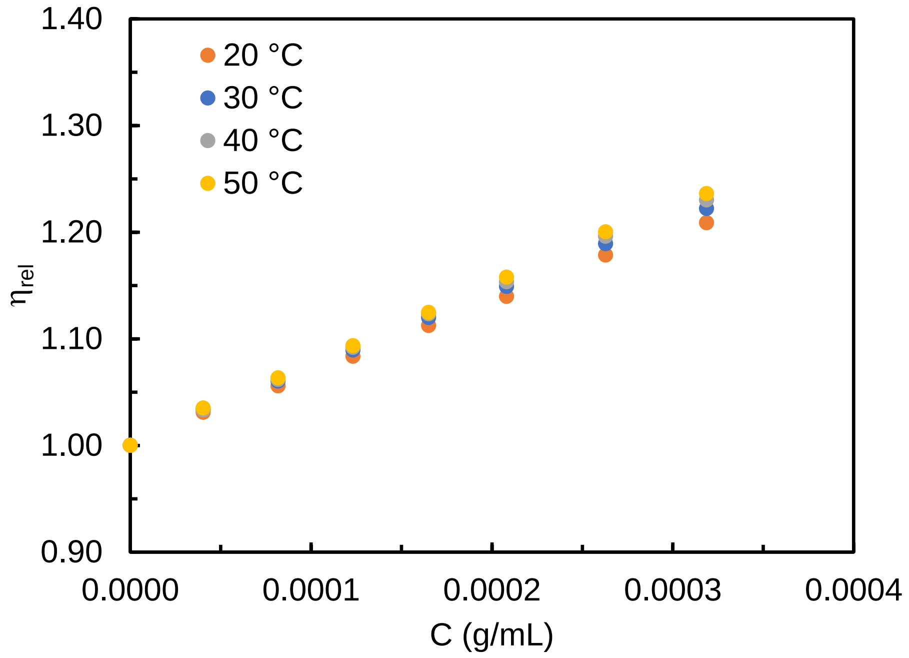

Figure S5. Xanthan gum relative viscosity as a function of xanthan gum concentration in 1.14 mM $CaCl_2 \times 2H_2O$ at varied temperatures.

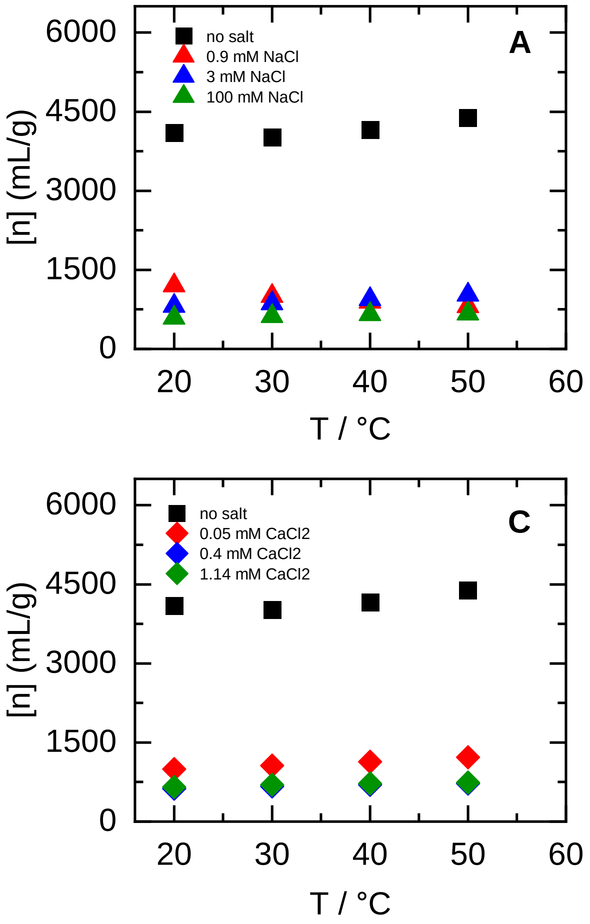

Figure S6. XG intrinsic viscosity in aqueous NaCl (**A**), KCl (**B**) and $CaCl_2$ (**C**). Panel **D** shows a comparative summary for similar salt concentrations. The error bars are relative standard deviations and within the symbol size.

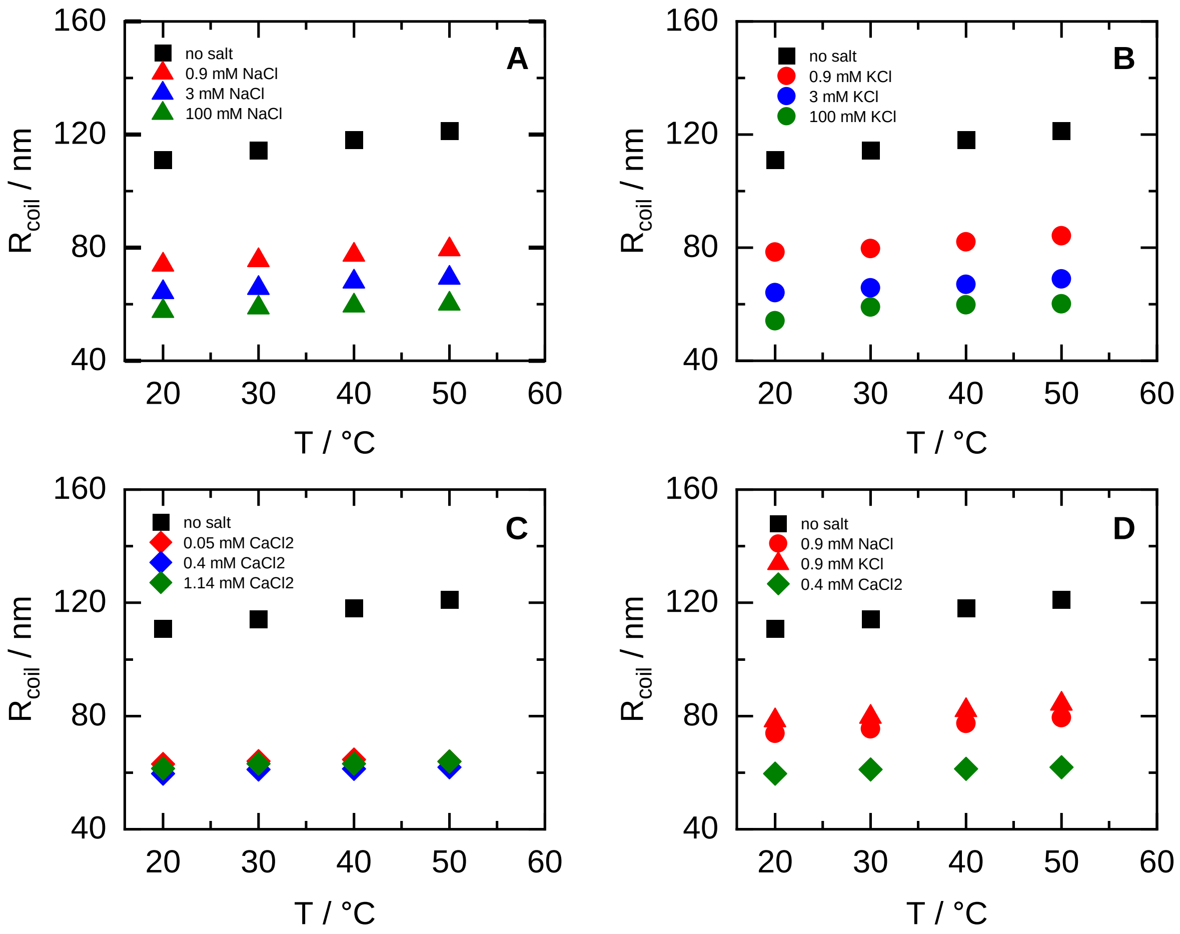

Figure S7. XG coil radius in aqueous NaCl (**A**), KCl (**B**) and $CaCl_2$ (**C**). Panel **D** shows a comparative summary for similar salt concentrations. The error bars are relative standard deviations and typically within the symbols.

Table S1. XG intrinsic viscosity, [η], with different NaCl concentrations as a function of temperatures.

		Huggins			Kraemer			Rao 1		Rao 2		Rao 3	
[NaCl] (mM)	T (°C)	[η] (mL/g)	R^2	K_H	[η] (mL/g)	R^2	K_K	[η] (mL/g)	R^2	[η] (mL/g)	R^2	[η] (mL/g)	R^2
0	20	4302	0.634	-0.03	4029	0.982	-0.29	4096	0.999	2587	0.980	1708	0.934
	30	4708	0.869	-0.10	4329	0.978	-0.31	4018	0.997	2539	0.970	1675	0.917
	40	5195	0.840	-0.12	4692	0.987	-0.31	4156	0.990	2589	0.956	1688	0.897
	50	5613	0.879	-0.12	5022	0.980	-0.30	4380	0.990	2674	0.952	1714	0.888
0.9	20	1271	0.529	-0.08	1258	0.963	-0.45	1201	0.999	1039	1.000	906	0.997
	30	1356	0.683	-0.12	1340	0.957	-0.47	999	0.997	1079	1.000	1173	0.996
	40	1462	0.834	-0.09	1442	0.985	-0.44	890	0.990	1157	1.000	1518	0.996
	50	1576	0.671	-0.07	1553	0.979	-0.42	810	0.990	1241	1.000	1920	0.996
3.0	20	838	0.665	-0.12	833	0.963	-0.52	810	0.999	720	0.998	642	0.994
	30	894	0.734	-0.12	888	0.957	-0.51	863	0.997	761	0.998	675	0.993
	40	989	0.812	-0.16	981	0.985	-0.53	941	0.990	822	0.998	721	0.992
	50	1049	0.697	-0.06	1041	0.979	-0.45	1027	0.990	887	0.997	771	0.992
100	20	598	0.488	-0.07	596	0.987	-0.50	589	0.999	539	0.999	495	0.997
	30	640	0.708	-0.13	638	0.983	-0.54	622	0.997	567	0.999	518	0.996
	40	663	0.312	-0.07	661	0.971	-0.49	653	0.990	593	0.999	540	0.996
	50	683	0.102	-0.09	681	0.796	-0.51	670	0.990	607	0.999	551	0.996

Table S2. XG intrinsic viscosity, [η], with different KCl concentrations as a function of temperatures.

		Huggins			Kraemer			Rao 1		Rao 2		Rao 3	
[KCl] (mM)	T (°C)	[η] (mL/g)	R^2	K_H	[η] (mL/g)	R^2	K_K	[η] (mL/g)	R^2	[η] (mL/g)	R^2	[η] (mL/g)	R^2
0	20	4302	0.634	-0.03	4029	0.982	-0.29	4096	0.999	2587	0.980	1708	0.934
	30	4708	0.869	-0.10	4329	0.978	-0.31	4018	0.997	2539	0.970	1675	0.917
	40	5195	0.840	-0.12	4692	0.987	-0.31	4156	0.990	2589	0.956	1688	0.897
	50	5613	0.879	-0.12	5022	0.980	-0.30	4380	0.990	2674	0.952	1714	0.888
0.9	20	1521	0.653	-0.20	1496	0.914	-0.52	1384	0.999	1142	0.995	950	0.984
	30	1592	0.748	-0.20	1563	0.938	-0.51	1441	0.997	1181	0.994	977	0.982
	40	1738	0.688	-0.19	1703	0.920	-0.49	1572	0.990	1268	0.994	1034	0.981
	50	1884	0.643	-0.19	1843	0.904	-0.48	1699	0.990	1350	0.994	1086	0.980
3.0	20	828	0.172	-0.15	825	0.774	-0.55	807	0.999	717	0.999	639	0.997
	30	895	0.360	-0.22	890	0.819	-0.60	851	0.997	751	0.998	666	0.996
	40	947	0.076	-0.08	942	0.787	-0.48	936	0.990	817	0.999	717	0.996
	50	1033	0.057	-0.08	1028	0.731	-0.48	1025	0.990	885	0.999	769	0.996
100	20	501	0.756	0.76	501	0.198	0.20	556	0.999	512	0.999	473	0.998
	30	647	0.507	-0.42	644	0.783	-0.80	598	0.997	547	0.999	501	0.997
	40	673	0.469	-0.41	670	0.761	-0.78	621	0.990	566	0.998	517	0.996
	50	686	0.439	-0.42	683	0.734	-0.79	633	0.990	576	0.999	525	0.997

Table S3. XG intrinsic viscosity, [η], with different $CaCl_2 \cdot 2H_2O$ concentrations as a function of different temperatures.

		Huggins			Kraemer			Rao 1		Rao 2		Rao 3	
[$CaCl_2$] (mM)	T (°C)	[η] (mL/g)	R^2	K_H	[η] (mL/g)	R^2	K_K	[η] (mL/g)	R^2	[η] (mL/g)	R^2	[η] (mL/g)	R^2
0	20	4302	0.634	-0.03	4029	0.982	-0.29	4096	0.999	2587	0.980	1708	0.934
	30	4708	0.869	-0.10	4329	0.978	-0.31	4018	0.997	2539	0.970	1675	0.917
	40	5195	0.840	-0.12	4692	0.987	-0.31	4156	0.990	2589	0.956	1688	0.897
	50	5613	0.879	-0.12	5022	0.980	-0.30	4380	0.990	2674	0.952	1714	0.888
0.05	20	791	0.729	1.10	796	0.346	0.42	996	0.999	869	0.996	761	0.994
	30	830	0.787	1.14	837	0.432	0.44	1065	0.997	921	0.996	801	0.994
	40	851	0.813	1.31	860	0.520	0.55	1132	0.990	972	0.996	839	0.994
	50	824	0.893	1.94	840	0.742	0.98	1222	0.990	1039	0.996	890	0.995
0.4	20	668	0.439	-0.32	665	0.801	-0.70	626	0.999	571	0.997	521	0.995
	30	722	0.459	-0.38	718	0.772	-0.74	666	0.997	603	0.998	548	0.995
	40	728	0.160	-0.18	725	0.668	-0.58	700	0.990	631	0.997	571	0.994
	50	746	0.202	-0.15	742	0.780	-0.55	720	0.990	648	0.997	585	0.994
1.14	20	731	0.516	-0.48	727	0.757	-0.83	658	0.999	596	0.997	542	0.994
	30	795	0.581	-0.54	789	0.777	-0.87	698	0.997	629	0.997	568	0.993
	40	795	0.579	-0.39	790	0.830	-0.74	725	0.990	651	0.997	587	0.994
	50	824	0.508	-0.44	818	0.763	-0.78	742	0.990	664	0.998	597	0.994
